# Cytokine and chemokine networks in colorectal cancer

**DOI:** 10.3389/fimmu.2026.1822271

**Published:** 2026-05-20

**Authors:** Xuhui Zhang, Ying Zhang, Rong Wang

**Affiliations:** 1 College of Traditional Chinese Medicine, Shandong University of Traditional Chinese Medicine, Jinan, Shandong, China; 2 College of Integrated Chinese and Western Medicine, Jining Medical University, Jining, Shandong, China

**Keywords:** biomarkers, chemokines, colorectal cancer, cytokines, epithelial–mesenchymal transition, inflammation, metastasis, tumor microenvironment

## Abstract

Chronic inflammation is a central driver of colorectal cancer initiation and progression, with cytokines and chemokines orchestrating key tumor-promoting processes within the tumor microenvironment. Accumulating evidence indicates that inflammatory mediators—including CCL2, CCL3, CCL5, CXCL1, CCL20, TNF-α, IL-6, IL-8, and TGF-β—contribute to tumor growth, metastasis, immune modulation, and therapeutic resistance. Chemokines regulate immune cell recruitment and stromal remodeling, thereby shaping tumor–immune interactions and influencing disease outcome. Pro-inflammatory cytokines such as TNF-α and IL-6 activate NF-κB and STAT3 signaling pathways, promoting cell survival, angiogenesis, and epithelial–mesenchymal transition (EMT). IL-8 enhances angiogenesis and neutrophil infiltration, while CCL20 and its related signaling networks support cancer stemness and metastatic dissemination. In contrast, TGF-β exhibits context-dependent dual functions, acting as a tumor suppressor in early stages but promoting immune evasion, EMT, and metastasis in advanced disease. This review summarizes the roles of cytokines and chemokines in colorectal cancer progression and highlights their potential as diagnostic biomarkers and therapeutic targets. A deeper understanding of cytokine-mediated signaling networks may provide new opportunities for precision therapy and improved clinical outcomes.

## 1 Introduction

Colorectal cancer remains a leading cause of cancer-related mortality worldwide, with clinical outcomes frequently constrained by late-stage diagnosis, high recurrence rates, and the emergence of therapeutic resistance (1–3). Although surgical intervention and conventional systemic therapies have improved survival for localized disease, patients with advanced or metastatic colorectal cancer continue to face limited targeted options and suboptimal prognoses (4–6). Current clinical biomarkers lack the sensitivity and dynamic range required for robust early detection, longitudinal disease monitoring, and precise risk stratification (7). Consequently, there is a pressing need to decipher the molecular architectures that drive colorectal cancer pathogenesis, particularly those mechanisms that bridge chronic inflammation with malignant transformation, immune evasion, and treatment failure (8, 9).

Chronic intestinal inflammation is a well-established cornerstone of colorectal cancer initiation and progression, fundamentally shaping the cellular and molecular landscape of the tumor microenvironment (TME) (10, 11). Within this ecosystem, cytokines and chemokines function as central conductors of intercellular communication, orchestrating a highly integrated signaling network that coordinates epithelial proliferation, stromal remodeling, angiogenesis, and leukocyte recruitment (12, 13). Pro-inflammatory cytokines, notably TNF-α, IL-6, IL-8, and TGF-β, converge on master transcriptional regulators such as NF-κB and STAT3 to sustain oncogenic signaling, promote EMT, and foster a survival-adaptive niche (14–16). Concurrently, chemokine gradients (CCL2, CCL5, CXCL1, CCL20) direct the spatial (17) trafficking of immune and stromal cells, thereby sculpting an immunosuppressive TME that shields tumor cells from immune surveillance and facilitates metastatic dissemination (14).

Many cytokines and chemokines exhibit stage-specific duality, transitioning from tumor-suppressive or immunostimulatory roles during early carcinogenesis to potent drivers of therapy resistance and aggressive disease in advanced stages (18, 19). This functional pleiotropy, coupled with extensive cross-talk between cytokine and chemokine axes, complicates therapeutic targeting and biomarker validation (12, 14). Despite accumulating evidence implicating specific inflammatory networks in colorectal cancer progression, a comprehensive, mechanism-driven synthesis that links their molecular functions to clinical translation remains fragmented (11, 20). Moreover, the precise integration of these networks with gut-specific factors—such as microbiota-host interactions and mucosal barrier dynamics—requires systematic elucidation to unlock their full diagnostic and therapeutic potential (21–23). To address these critical knowledge gaps, this review summarizes recent advances in the understanding of cytokine and chemokine networks in colorectal cancer, providing a cohesive framework that bridges fundamental inflammatory biology with precision oncology, and offering a roadmap for biomarker-guided interventions in colorectal cancer.

## 2 Chemokines in colorectal cancer progression

### 2.1 CCL2 and CCL3 in colorectal cancer

CCL2 signals primarily through its cognate receptor CCR2, which is constitutively expressed on circulating classical monocytes (24). In colorectal cancer, CCL2 is markedly upregulated in both tumor epithelial cells and stromal compartments, particularly cancer-associated fibroblasts (CAFs) and tumor-associated macrophages (TAMs) (25, 26). Clinical cohorts have consistently demonstrated elevated CCL2 expression in colorectal cancer tissues relative to adjacent normal mucosa, with levels correlating with advanced TNM stage, lymph node metastasis, and reduced disease-free survival (27, 28). CCL2 secretion establishes a chemotactic gradient that recruits CCR2^+^ monocytes into the TME. Upon infiltration, CCL2–CCR2 engagement activates canonical PI3K/AKT and JAK/STAT3 signaling, promoting monocyte survival, metabolic reprogramming, and differentiation toward an M2-like TAM phenotype. These M2-polarized macrophages, in turn, secrete anti-inflammatory cytokines (IL-10, TGF-β), pro-angiogenic factors (VEGF), and matrix-remodeling enzymes (MMP-9), collectively fostering an immunosuppressive, pro-invasive niche (25). Notably, CCL2-driven macrophage recruitment has been implicated in the establishment of pre-metastatic niches in the liver, a predominant site of colorectal cancer dissemination (29). From a translational perspective, dual CCL2-secreting populations—CAFs and TAMs—represent promising cellular sources for liquid or tissue-based biomarker development, while pharmacologic disruption of the CCL2–CCR2 axis is under active investigation as a strategy to reprogram the TME and sensitize tumors to immunotherapy (30, 31).

In contrast to the predominantly tumor-promoting role of CCL2, CCL3 exhibits functional pleiotropy shaped by microenvironmental cues and disease stage. CCL3 signals through a receptor repertoire that includes CCR1, CCR4, and CCR5, enabling recruitment of diverse leukocyte subsets, including CD8^+^ T cells, NK cells, dendritic cells (DCs), and subsets of B cells (32–34). In experimental models of colitis-associated colorectal cancer (CAC), CCL3-mediated chemotaxis facilitates the infiltration of antigen-presenting cells and cytotoxic lymphocytes, thereby contributing to early immune surveillance and tumor containment (35). However, within established tumors, chronic inflammation and immunosuppressive signals can repurpose CCL3 signaling to support myeloid-derived suppressor cell (MDSC) expansion and regulatory T-cell (Treg) recruitment, inadvertently reinforcing immune evasion (36). Pharmacologic studies further underscore the therapeutic relevance of CCL3 modulation: imipramine, a tricyclic antidepressant with GPCR-targeting properties, attenuates colorectal cancer progression in part by suppressing CCL3 production and disrupting downstream chemokine-mediated signal transduction (35, 37). Emerging evidence suggests that CCL2 and CCL3 do not operate in isolation but engage in cooperative or competitive crosstalk with other chemokines (CCL5, CXCL1) to shape the immune architecture of colorectal cancer. For instance, CCL2-driven TAM accumulation can amplify local CCL3 production through paracrine IL-6/STAT3 signaling, creating a feed-forward loop that sustains myeloid cell recruitment (25, 26). Conversely, CCL3-mediated DC activation may partially counterbalance CCL2-induced immunosuppression by enhancing cross-presentation of tumor antigens (38, 39). Deciphering these dynamic interactions, particularly their spatial organization within distinct TME sites, will be critical for designing precision interventions that selectively disrupt pro-tumorigenic chemokine circuits while preserving antitumor immunity.

### 2.2 CCL5 and CXCL1 in colorectal cancer

The chemokine CCL5 is involved in the chemotaxis of T lymphocytes, eosinophils, and basophils. In tissue samples from patients with colorectal cancer, CCL5 levels were also markedly higher than those in normal colorectal tissues (17). Compared with healthy controls, patients with colorectal cancer exhibited elevated blood levels of CCL5, which increases the potential utility of CCL5 as a diagnostic biomarker for colorectal cancer (40). One study showed that patients with elevated CCL5 expression had a lower 5-year survival rate, indicating that CCL5 may serve not only as an important diagnostic marker but also as a prognostic biomarker associated with colorectal cancer (41). The chemokine CXCL1, also known as neutrophil-activating protein 3 (NAP-3), is a chemoattractant for neutrophils (42). In patients with colorectal cancer, dendritic cells exhibit elevated levels of CXCL1, which promotes colorectal cancer cell metastasis by increasing the expression of MMP-7 and supporting the induction of EMT (43). However, some studies have shown that although CXCL1 levels are upregulated in tumor tissues, CXCL1 expression tends to decline as the tumor progresses. Analyses further indicate that colorectal cancer patients with high CXCL1 expression have longer overall survival and a better prognosis (44). Therefore, CXCL1 may currently serve as an auxiliary biomarker for the diagnosis of colorectal cancer. Nevertheless, because its expression level is negatively correlated with tumor progression, the precise role of CXCL1 in the occurrence and development of colorectal cancer requires further investigation. Moreover, CCL2, CCL3, CCL5, and CXCL1 form an interconnected chemokine network that reshapes the colorectal cancer tumor microenvironment (16, 45). CCL2 promotes the recruitment of CCR2^+^ monocytes and their differentiation into tumor-associated macrophages, whereas CCL5 facilitates the accumulation of immunosuppressive myeloid cells and regulatory lymphocyte populations (46). CXCL1 mainly drives neutrophil infiltration and may support the expansion of myeloid-derived suppressor cells, thereby enhancing extracellular matrix remodeling, angiogenesis, and tumor invasion (47, 48). Although CCL3 can recruit dendritic cells, NK cells, and lymphocytes, its function may become context-dependent within chronic inflammation (36). Together, these chemokines coordinate immune-cell trafficking, stromal activation, and inflammatory remodeling, thereby creating a tumor-permissive niche that supports colorectal cancer progression and metastasis.

### 2.3 CCL20 in colorectal cancer

The transcriptional induction of CCL20 within the colon tumor microenvironment is tightly coupled to cytokine-driven and microbial-sensing pathways (49). Pro-inflammatory mediators, particularly IL-6 and TNF-α, robustly upregulate CCL20 expression via STAT3 and NF-κB activation (50). Concurrently, Toll-like receptors (TLRs) serve as critical environmental sensors; in HT29 colorectal cancer cells, TLR3 engagement triggers robust CCL20 transcription and secretion, whereas TLR3 silencing markedly attenuates both mRNA and protein output (49, 51). TLR4 signaling similarly contributes to CCL20 production, underscoring the role of pathogen- and damage-associated molecular patterns in sustaining chemokine output (52). Downstream of CCL20–CCR6 engagement, activation of PI3K/AKT, ERK1/2, and p38 MAPK cascades sustains oncogenic signaling, promotes survival under metabolic stress, and reinforces a feed-forward inflammatory loop that amplifies local cytokine production and stromal activation (53). CCL20–CCR6 signaling enhances the self-renewal and chemoresistance of tumor-initiating cells, thereby fueling disease recurrence following conventional therapy. Mechanistically, CCL20 cooperates with Wnt/β-catenin and TGF-β pathways to upregulate EMT-associated transcription factors (SNAIL, TWIST), suppress E-cadherin expression, and remodel the extracellular matrix, collectively facilitating invasive dissemination (54, 55). *In vivo* murine models corroborate these findings, demonstrating that CCL20 overexpression accelerates liver and peritoneal metastasis, whereas CCR6 blockade or genetic ablation significantly impairs metastatic colonization (56, 57). These data position the CCL20–CCR6 axis as a critical node linking inflammatory signaling to stem-like plasticity and metastatic competence.

**Figure 1:**
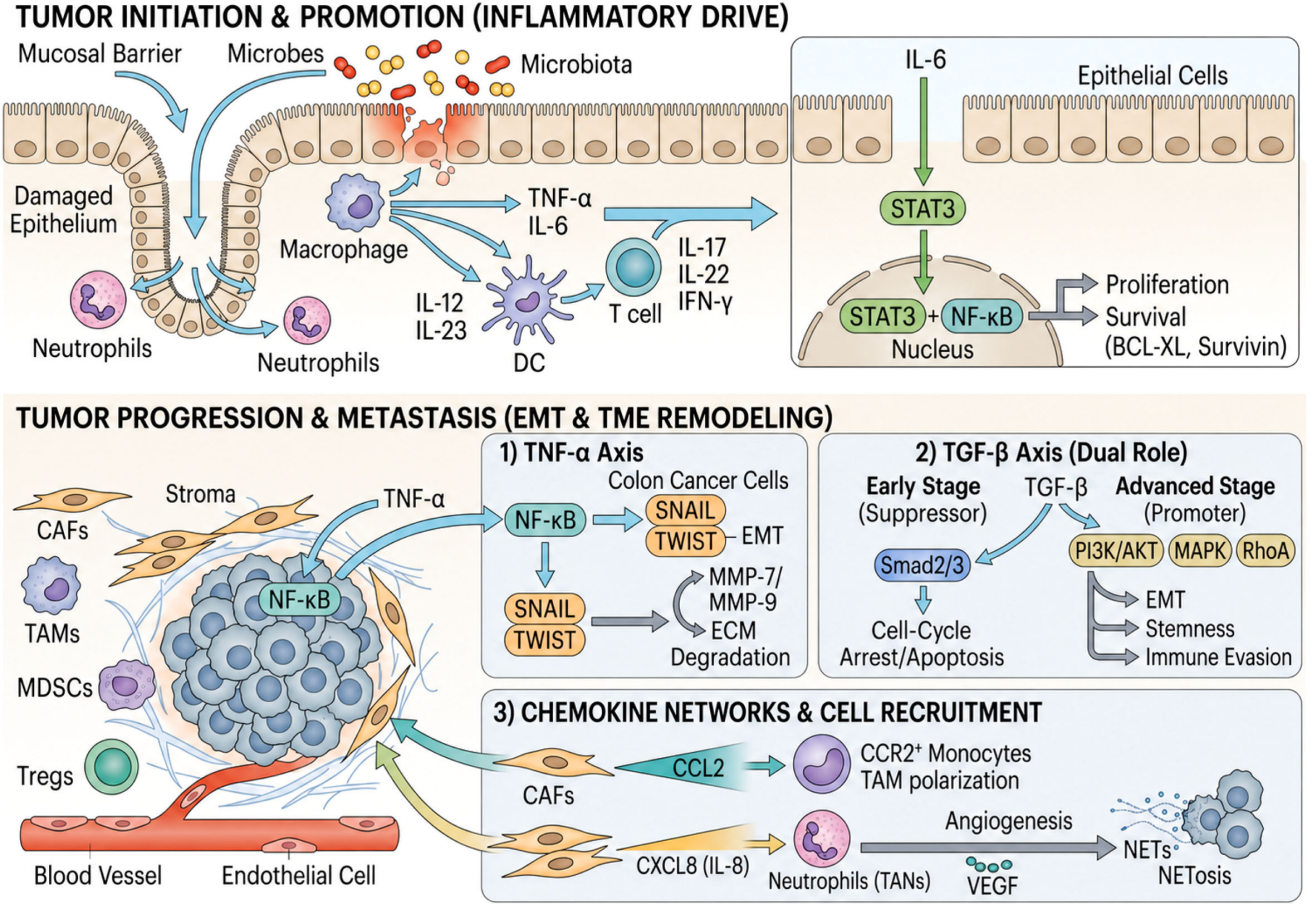
Cytokine and chemokine networks in colorectal cancer.

Under conditions of sustained inflammation, CCL20 drives macrophage polarization toward an immunosuppressive M2 phenotype and facilitates the recruitment of regulatory T cells, thereby shielding tumors from cytotoxic immunity (54). Conversely, CCL20’s high affinity for CCR6 on immature dendritic cells can be therapeutically harnessed to enhance antigen cross-presentation and initiate adaptive immune responses (58). In syngeneic CT26 colorectal cancer models, intratumoral administration of recombinant CCL20 or CCL20-expressing viral vectors promotes robust DC infiltration, triggers tumor-specific cytotoxicity, and induces significant regression (14, 59, 60). Similarly, dietary vitamin D3 suppresses azoxymethane/dextran sulfate sodium (AOM/DSS)-induced tumorigenesis by downregulating CCL20 and inhibiting p38MAPK–NF-κB signaling, illustrating how microenvironmental modulation can flip CCL20 from a driver to a suppressor of malignancy (61). (**Figure 1**).

## 3 Cytokines in colorectal cancer

### 3.1 TNF-α in colorectal cancer

TNF is an important factor capable of inducing apoptosis in tumor cells, and it can trigger apoptosis in various tumor cell types *in vitro*. However, studies have shown that the principal role of TNF-α *in vivo* is not tumoricidal; rather, it acts as an inflammatory mediator that promotes tumor progression (62, 63). High TNF-α expression has been detected in a large number of patients with colorectal cancer, and its level may rapidly return to normal after resection of the primary tumor lesion. One possible explanation is that colorectal cancer stimulates the host immune system, thereby elevating TNF-α levels, whereas surgical removal of the tumor interrupts or reduces tumor-mediated stimulation of lymphocytes, resulting in decreased TNF-α production by these cells (18, 64). Collectively, these observations indicate a strong association between TNF-α and colorectal cancer. In a mouse model of colitis-associated colorectal cancer, Popivanova et al. (65) found that TNF-α expression was substantially increased in the submucosa and lamina propria of colorectal tumors induced by azoxymethane (AOM) and dextran sulfate sodium (DSS). In mice deficient in tumor necrosis factor receptor I (TNFRI), the incidence of colorectal cancer was significantly reduced, and intravenous infusion of neutralizing antibodies to block TNF-α signaling also significantly reduced the incidence of colorectal cancer and inhibited tumor growth (18, 66). TNF-α and other early pro-inflammatory mediators, such as IL-6, in patients with colorectal cancer are mainly produced by macrophages and dendritic cells in the colorectalic lamina propria (67, 68). The tumor-promoting effects of TNF-α are mediated primarily through activation of two key inflammatory signaling pathways, nuclear factor-κB (NF-κB) and activator protein-1 (AP-1) (18, 69), thereby initiating the expression of pro-angiogenic factors such as IL-8 and matrix metalloproteinases (MMPs), and upregulating the binding of the adhesion molecule CD44v6 to its ligand hyaluronic acid, which promotes tumor implantation/colorectalization (70).

In addition, the pro-metastatic effects of TNF-α are closely linked to its activation of NF-κB signaling, which directly contributes to the stabilization and functional activation of the EMT-associated transcription factor SNAIL (71, 72). NF-κB activation enhances SNAIL transcription and inhibits its proteasomal degradation, thereby sustaining its nuclear accumulation (73). Stabilized SNAIL represses epithelial markers such as E-cadherin while promoting mesenchymal gene expression, facilitating epithelial–mesenchymal transition and enhancing tumor cell invasiveness (74, 75). NF-κB–dependent inflammatory signaling cooperates with TNF-α–induced pathways to maintain a pro-inflammatory microenvironment that further reinforces EMT and metastatic dissemination (71). This integrated TNF-α/NF-κB/SNAIL axis provides a mechanistic link between chronic inflammation and colorectal cancer progression. Given the role of TNF-α-mediated colorectal inflammatory effects in tumor progression, TNF-α-blocking agents are already available clinically and are used for the treatment of colorectal cancer-associated inflammatory conditions, including Crohn’s disease and ulcerative colitis (18, 76) (**Supplementary Table 1**).

### 3.2 Interleukin in colorectal cancer

#### 3.2.1 IL-6 in colorectal cancer

IL-6 and IL-8 are both key cytokines involved in the progression of colorectal cancer, and their expression levels in advanced colorectal cancer are markedly higher than those in normal tissues (77, 78). In the early stage, IL-6 is produced mainly by macrophages and dendritic cells in the lamina propria, whereas in later stages it is released by T cells (79, 80). In both APC^Min^/^+^ colorectal cancer mouse models and colitis-associated colorectal cancer models, IL-6 has been shown to promote the proliferation of transformed cells at an early stage, as well as tumor cell growth (81, 82). Peripheral blood IL-6 levels in patients with colorectal cancer are closely associated with clinical stage and disease progression (83, 84), and IL-6 is substantially elevated in patients with advanced disease and late-stage liver metastasis (85, 86). Colorectal cancer cells themselves can hypersecrete IL-6, which primarily stimulates colorectal cancer cell proliferation through activation of the downstream transcription factor Stat3 (87). This is accompanied by sustained activation of NF-κB, which promotes the survival of colorectal cancer cells and resistance to externally induced apoptosis (88). NF-κB activation in tumor and immune cells induces the transcription of cytokines such as IL-6 and IL-11, which subsequently activate STAT3 signaling in an autocrine and paracrine fashion (89). In turn, STAT3 enhances the expression of genes involved in cell survival (BCL-XL, survivin), proliferation (cyclin D1), and immune suppression, while also sustaining NF-κB activity through feedback regulation of inflammatory mediators (90, 91).

Beyond its direct proliferative effects, IL-6 exerts profound immunosuppressive and tumor-promoting functions within the colorectal cancer microenvironment. IL-6 signaling promotes the expansion and recruitment of MDSCs, which inhibit cytotoxic T cell activity through arginase-1 production, nitric oxide synthesis, and ROS generation (92, 93). In parallel, IL-6 drives the polarization of macrophages toward an M2-like tumor-associated phenotype, characterized by increased secretion of IL-10 and TGF-β, thereby reinforcing immune suppression and tissue remodeling (26, 94). Furthermore, IL-6 contributes to T cell dysfunction and exhaustion by upregulating inhibitory receptors and impairing effector cytokine production (95). At the stromal level, IL-6 activates CAFs, enhancing extracellular matrix deposition and creating a physical and biochemical barrier to immune cell infiltration (96). Therefore, these IL-6–mediated effects establish an immunosuppressive and pro-tumorigenic inflammatory niche that facilitates colorectal cancer progression.

#### 3.2.2 IL-8 in colorectal cancer

IL-8, systematically designated as CXCL8, functions as a master pro-angiogenic and pro-inflammatory chemokine that plays a central role in colorectal cancer progression. Its expression is tightly correlated with advanced TNM stage, venous invasion, and reduced overall survival, positioning it as both a robust prognostic indicator and a functional driver of malignant evolution (97, 98). CXCL8 signals primarily through G protein-coupled receptors CXCR1 and CXCR2, activating downstream PI3K/AKT, RAS/MAPK, and NF-κB cascades that collectively sustain tumor cell survival, stromal remodeling, and immune evasion (99). Within the colon tumor microenvironment, CXCL8 directly stimulates endothelial cell proliferation, migration, and vascular permeability, thereby accelerating neovascularization and facilitating nutrient delivery to hypoxic tumor cores (100, 101). This angiogenic program is frequently amplified by hypoxia-inducible factor 1α (HIF-1α), which transcriptionally upregulates CXCL8 in both malignant epithelial cells and CAFs (102, 103). Besides, CXCL8 exerts potent autocrine and paracrine mitogenic effects on colorectal cancer cells, sustaining MAPK-driven proliferative signaling and conferring resistance to DNA-damaging chemotherapeutics and apoptosis-inducing stressors (104–106).

Beyond vascular remodeling, CXCL8 critically shapes the invasive front by upregulating matrix metalloproteinases, particularly MMP-2 and MMP-9 (historically termed type IV collagenases) (104, 107). These enzymes degrade basement membrane and interstitial matrix components, dismantling tissue architecture and facilitating local invasion, intravasation, and distant dissemination (108). Furthermore, CXCL8 serves as the principal chemoattractant for neutrophils within the colon TME. Recruited neutrophils frequently adopt a tumor-associated neutrophil (TAN) phenotype, characterized by enhanced secretion of gelatinases, proteases, and ROS (109). In advanced disease, TANs can undergo NETosis, releasing neutrophil extracellular traps (NETs) that capture circulating tumor cells, remodel pre-metastatic niches (particularly in the liver), and shield malignant clones from cytotoxic lymphocyte surveillance (110–112). The tumor-promoting activities of CXCL8 do not operate in isolation but synergize with parallel inflammatory axes that collectively drive colon carcinogenesis (113). Persistent CXCL8 signaling amplifies local ROS/RNS production, which induces oxidative DNA lesions, strand breaks, and elevated mutational burden (114). It also intersects with the COX-2/PGE_2_ axis, which stabilizes β-catenin and enhances Wnt-driven epithelial proliferation, while inflammasome activation (NLRP3) promotes IL-1β maturation that further upregulates CXCL8 transcription via NF-κB (115, 116). Together, these pathways establish a self-reinforcing inflammatory ecosystem that couples genomic instability with epigenetic remodeling, including aberrant DNA methylation and histone modification, ultimately silencing tumor suppressor loci and locking colorectal cancer into an aggressive, therapy-resistant phenotype.

#### 3.2.3 Other interleukins in colorectal cancer

Beyond IL-6 and IL-8, the IL-23/IL-17/IL-22 axis represents a particularly relevant inflammatory circuit in colorectal cancer because it links mucosal barrier damage, microbiota-driven immune activation, and epithelial regeneration (117, 118). Following epithelial injury, antigen-presenting cells in the lamina propria can produce IL-23, which promotes IL-17 and IL-22 secretion by Th17 cells, γδ T cells, and innate lymphoid cells. IL-17 enhances neutrophil recruitment, stromal activation, and inflammatory cytokine amplification, whereas IL-22 acts directly on intestinal epithelial cells to promote proliferation, survival, antimicrobial peptide expression, and tissue repair (119–121). Although these responses are protective during acute mucosal injury, persistent activation may favor aberrant crypt regeneration, expansion of transformed epithelial clones, and colitis-associated carcinogenesis (117, 122). IL-22 activates STAT3-dependent transcriptional programs in epithelial cells, supporting anti-apoptotic signaling, stem-like cell maintenance, and regenerative hyperplasia. Thus, in colorectal cancer, cytokines can promote malignancy not only by suppressing antitumor immunity but also by reshaping the epithelial repair program that governs crypt homeostasis.

IL-33/ST2 signaling provides another colon-specific example of how inflammatory cytokines may exert context-dependent effects. IL-33 is released as an alarmin from damaged epithelial and stromal cells after mucosal injury (123). Through its receptor ST2, IL-33 can activate group 2 innate lymphoid cells, mast cells, macrophages, and regulatory T cells, thereby promoting tissue repair and limiting acute epithelial damage (124–126). However, sustained IL-33/ST2 activation may also create a tumor-permissive microenvironment by enhancing epithelial proliferation, angiogenic mediator production, myeloid-cell recruitment, and immunoregulatory cell expansion (127). This dual effect is particularly relevant to colorectal cancer, where repeated epithelial injury and repair cycles can transform wound-healing inflammation into malignant regeneration.

### 3.3 TGF-β in colorectal cancer

TGF-β represents a paradigmatic example of cytokine pleiotropy in oncology, functioning as a critical gatekeeper in early carcinogenesis while evolving into a master driver of advanced colorectal cancer (128, 129). In normal colonic epithelium and early adenomatous lesions, TGF-β exerts potent tumor-suppressive effects by enforcing cell-cycle arrest, promoting apoptosis, and maintaining genomic stability through canonical Smad2/3-dependent signaling (130). Genetic disruption of this axis, such as *Smad3* inactivation, predisposes murine models to spontaneous intestinal tumorigenesis, underscoring its non-redundant role in restraining neoplastic transformation (131). However, as colorectal cancer progresses, malignant cells frequently acquire resistance to TGF-β-mediated cytostasis through somatic mutations in *TGFBR2*, *SMAD4*, or upstream regulators, effectively decoupling the growth-inhibitory arm of the pathway (132, 133).

Loss of canonical TGF-β responsiveness triggers a functional transition that repurposes the cytokine into a potent tumor-promoting signal. In established tumors, dysregulated TGF-β engages non-canonical cascades (PI3K/AKT, TAK1/p38 MAPK, RhoA/ROCK) that synergize with oncogenic mutations to enhance cell survival, metabolic adaptation, and therapy resistance (134, 135). Critically, TGF-β orchestrates profound immunosuppression within the colon tumor microenvironment by inhibiting cytotoxic CD8^+^ T cell and NK cell effector functions, impairing dendritic cell maturation, and expanding Treg and MDSC populations (133). Murine models with T cell–specific *Smad4* deletion demonstrate accelerated gastrointestinal tumorigenesis, highlighting how TGF-β-mediated immune exclusion shields malignant clones from immunosurveillance (53, 136). Concurrently, TGF-β drives CAFs activation and excessive extracellular matrix (ECM) deposition, fostering a dense desmoplastic stroma that acts as a physical and biochemical barrier to drug penetration and immune cell infiltration (137).

Beyond immune evasion, TGF-β is a central inducer of EMT, a plasticity program essential for local invasion and hematogenous spread (138). By upregulating transcriptional repressors (SNAIL, SLUG, ZEB1/2) and downregulating junctional complexes (E-cadherin, claudins), TGF-β dismantles epithelial integrity and confers migratory, invasive, and stem-like properties to colorectal cancer cells (139–141). This EMT program is frequently amplified by inflammatory crosstalk; for instance, TNF-α and IL-6 stabilize EMT-associated transcription factors via NF-κB and STAT3, creating a synergistic loop that reinforces mesenchymal transition (142, 143). Additionally, TGF-β promotes tumor angiogenesis indirectly by inducing VEGF expression in both malignant and stromal compartments, while simultaneously remodeling the vascular niche to facilitate intravasation and liver-directed metastatic colonization (144). The convergence of EMT, desmoplasia, and angiogenic remodeling establishes a highly permissive niche for systemic dissemination.

## 4 Monomers target inflammatory cytokine networks in colorectal cancer

In colorectal cancer, inflammation-driven remodeling of the tumor microenvironment represents a central mechanism underlying tumor initiation and progression (145–147). Increasing evidence indicates that bioactive monomers derived from traditional medicinal compounds exert potent anti-tumor effects by targeting inflammatory cytokine networks in a multi-layered manner (148–152). Natural compounds such as flavonoids, alkaloids, and polyphenols have been shown to suppress the production of key pro-inflammatory cytokines, including IL-1β, IL-17, and IL-23, while inhibiting critical signaling cascades such as the NF-κB signaling pathway, STAT3 signaling pathway, and NLRP3 inflammasome (153–156). Curcumin attenuates chronic inflammation by reducing macrophage polarization toward the M2-macrophage and by restoring the balance between Th1 and regulatory T cells, thereby dampening the IFN-γ production (157–159). Notably, within the intestine-specific context, certain natural monomers can also modulate gut microbiota composition and metabolic outputs, such as short-chain fatty acids, which in turn reinforce epithelial barrier integrity and limit the translocation of microbial-associated molecular patterns that trigger inflammatory cascades (160–163). Accordingly, traditional-medicine-derived natural monomers may suppress inflammation-driven colorectal cancer progression by modulating TNF-α/NF-κB/VEGF signaling, microbiota-dependent cytokine production, macrophage polarization, and mucosal inflammatory remodeling, thereby providing a mechanistic basis for inflammation-targeted adjunctive therapy.

## 5 Conclusion

Cytokine and chemokine networks function as central conductors of colorectal cancer pathogenesis, integrating chronic inflammation with malignant transformation, immune evasion, and metastatic dissemination. As detailed throughout this review, key mediators—including CCL2, CXCL1, TNF-α, IL-6, IL-8, and TGF-β—orchestrate tumor-promoting processes by converging on master signaling axes such as NF-κB, STAT3, and TGF-β/Smad. These cascades collectively sustain cancer stemness, drive epithelial–mesenchymal transition, remodel stromal architecture, and foster an immunosuppressive niche. Critically, these mediators exhibit profound stage-specific duality, transitioning from early immunostimulatory functions to potent drivers of therapeutic resistance in advanced disease. Their strong correlations with clinical progression and survival underscore their value as robust diagnostic and prognostic biomarkers.

Despite substantial mechanistic advances, the precise spatiotemporal dynamics and context-dependent plasticity of these inflammatory circuits remain incompletely resolved. Elucidating how cytokine gradients intersect with gut-specific microenvironmental cues, including microbiota-host crosstalk and mucosal regeneration, is essential for clinical translation. Future research must integrate spatial transcriptomics, multi-omics profiling, and functional validation to map context-specific signaling networks with higher resolution. Therapeutically, precision interventions should prioritize stage-adapted, rational combination strategies that selectively dismantle pro-tumorigenic cytokine axes while preserving antitumor immunity. By bridging mechanistic insights with biomarker-driven clinical frameworks, targeting inflammatory networks will ultimately enable personalized therapeutic paradigms and improve long-term outcomes in colorectal cancer.
